# Bioengineered Probiotics: Synthetic Biology Can Provide Live Cell Therapeutics for the Treatment of Foodborne Diseases

**DOI:** 10.3389/fbioe.2022.890479

**Published:** 2022-05-17

**Authors:** Karla Cristina P. Cruz, Laura O. Enekegho, David T. Stuart

**Affiliations:** Department of Biochemistry, University of Alberta, Edmonton, AB, Canada

**Keywords:** probiotic, synthetic biology, biosensors, metabolic engineering, *Vibrio cholerae*, *Clostridium perfringens*, *Staphylococcus aureus*, *Clostridioides difficile*

## Abstract

The rising prevalence of antibiotic resistant microbial pathogens presents an ominous health and economic challenge to modern society. The discovery and large-scale development of antibiotic drugs in previous decades was transformational, providing cheap, effective treatment for what would previously have been a lethal infection. As microbial strains resistant to many or even all antibiotic drug treatments have evolved, there is an urgent need for new drugs or antimicrobial treatments to control these pathogens. The ability to sequence and mine the genomes of an increasing number of microbial strains from previously unexplored environments has the potential to identify new natural product antibiotic biosynthesis pathways. This coupled with the power of synthetic biology to generate new production chassis, biosensors and “weaponized” live cell therapeutics may provide new means to combat the rapidly evolving threat of drug resistant microbial pathogens. This review focuses on the application of synthetic biology to construct probiotic strains that have been endowed with functionalities allowing them to identify, compete with and in some cases kill microbial pathogens as well as stimulate host immunity. Weaponized probiotics may have the greatest potential for use against pathogens that infect the gastrointestinal tract: *Vibrio cholerae*, *Staphylococcus aureus*, *Clostridium perfringens* and *Clostridioides difficile*. The potential benefits of engineered probiotics are highlighted along with the challenges that must still be met before these intriguing and exciting new therapeutic tools can be widely deployed.

## 1 Introduction

The discovery and application of antibiotic drugs is among the most significant accomplishments of medical science. Alexander Fleming’s discovery of penicillin (Fleming, 1929) and subsequent discovery and development of multiple classes of natural product antibiotics have been transformational to modern society. These compounds have yielded cheap and effective treatments for diseases caused by common bacterial infections that would previously have proven fatal. The advent of effective antibiotic drugs has made it possible to survive complex surgical procedures like open heart surgery and organ transplants and extended the average human life-span (Riley, 2005; Kaviani et al., 2020). The benefits of readily available antibiotic drugs have extended into agriculture and aquaculture, making it possible to increase productivity of farmed animals (Park et al., 1994; Patel et al., 2020). Indeed, the application of prophylactic antibiotics was recognized as growth promoting for farm animals in the 1940s and this has had a significant economic benefit to the agriculture industry (Moore et al., 1946; Graham et al., 2007).

The first widely employed antibiotics introduced in the early 1900s were synthetic compounds identified by Paul Ehrlich. The compound arsphenamine was one of the first, employed as a treatment for syphilis (Ehrlich, 1913; Gelpi et al., 2015). This was followed by the development of sulfonamides (Domagk, 1935; Bickel, 1988). A variety of other synthetic compounds were subsequently identified through systematic screening efforts and these have been previously reviewed (Hutchings et al., 2019). The synthetic antibiotic drugs were largely displaced by penicillin and the natural product antibiotics that were subsequently identified and developed (Jones et al., 1944; Bryer et al., 1948; Duggar, 1948; Walksman and Lechevalier, 1949; Newton and Abraham, 1955). Most of the major classes of antibiotic drugs were discovered during the period from the 1950s to the 1970s which was considered a “golden era” for antibiotic drug development (Nicolaou and Rigol, 2018). Many of these including streptomycin, neomycin and tetracycline remain in use today and virtually all widely used antibiotics either are or are derived from natural products produced by soil bacteria and fungi, a testament to the chemical diversity explored by these organisms in their natural environments (Waksman et al., 2010). The full realm of natural product and synthetic antibiotic drugs has been extensively reviewed (Durand et al., 2019; Hutchings et al., 2019). Since that time there has been a decline in the discovery of natural product antibiotics and an increased reliance on the modification of existing drug compounds to respond to the rising antibiotic resistance of pathogens (Olsufyeva and Yankovskaya, 2020; Acharya et al., 2022). High throughput screening of chemical libraries has met with limited success in identification and development of new antibiotic drugs, reinforcing how powerful a force natural selection has been in generating antibiotic natural products in microbial cells (Chopra et al., 2002; Durand et al., 2019).

The decline in discovery of new antibiotic drugs has become an issue of increasing concern reflecting a rise in the frequency of infections caused by pathogens resistant to most or even all of the currently used antimicrobial drugs. It has been estimated that antibiotic resistant bacterial infections were responsible for greater than one million deaths in 2019 (Murray et al., 2022). It has further been projected, that deaths directly attributable to antibiotic resistant infections could reach 10 million per year by 2050 (Murray et al., 2022). The growing occurrence of antibiotic resistance poses not only a global health threat but a potential economic crisis that could cost up to $1 trillion per year by 2050 (O’Neil, 2016).

Bacterial strains with resistance to virtually all currently employed antibiotic drugs have been identified (Chang et al., 2003). Widespread use and misuse of antibiotic drugs for both humans and animals has been touted as a key factor in the development of antibiotic resistance (Ventola, 2015; Pulingam et al., 2022). However, given that most antibiotic drugs are produced by bacteria and fungi resident in soil, it is not surprising that resistant strains exist in the environment independent of clinical or agricultural exposure to antibiotics (D'Costa et al., 2006; Larsen et al., 2022). The answer to this ongoing problem lies in developing processes to identify and produce new antibiotic drugs and therapies. These processes need to keep pace with the ability of pathogens to acquire resistance to these measures.

## 2 Synthetic Biology Approaches to Antimicrobial Drug Resistance

**FIGURE 1:**
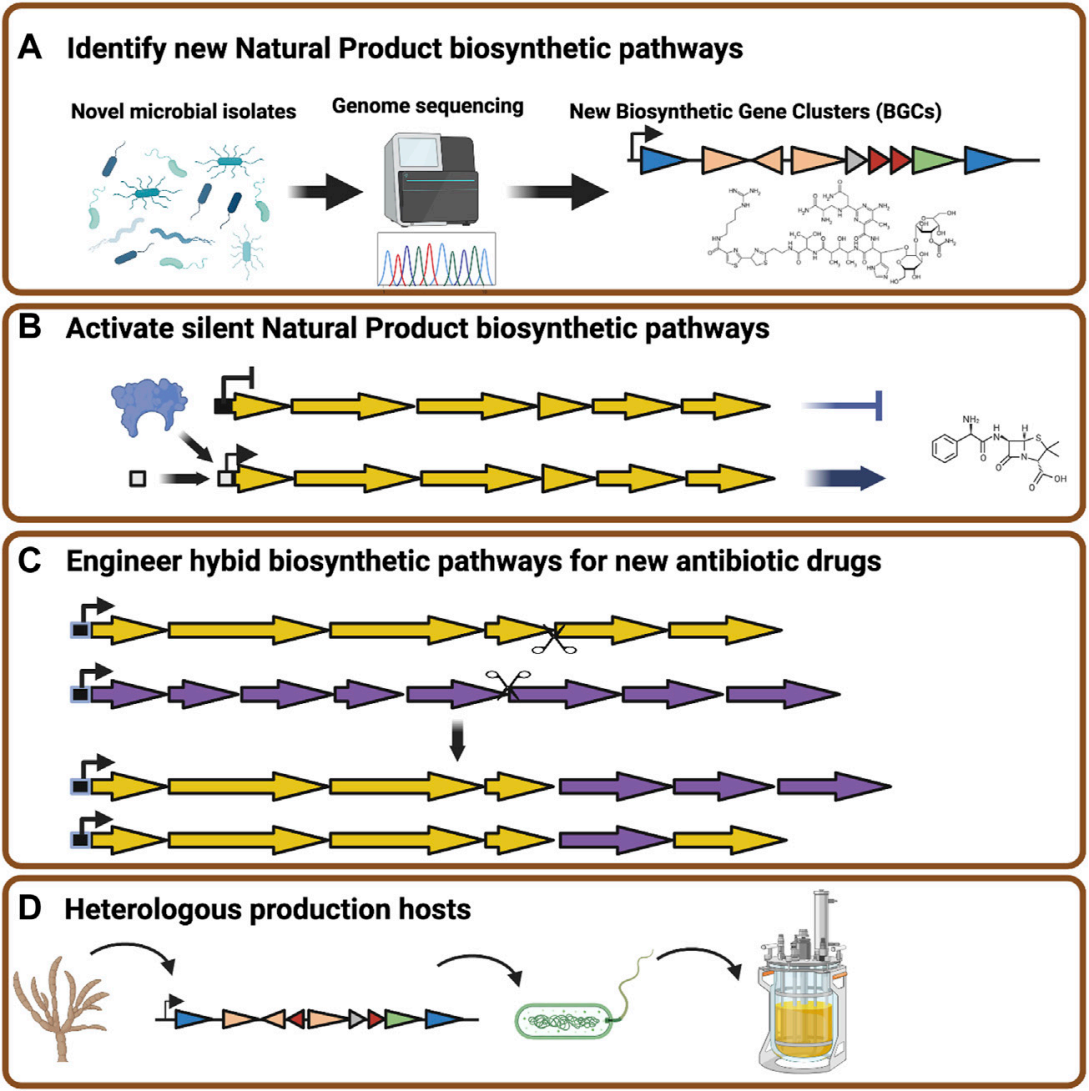
Strategies to expand the diversity of natural product antibiotics. **(A)** Rapid genome sequencing combined with bioinformatic tools can be applied to a broad range of bacteria and fungi isolated from unusual environments to identify natural product biosynthetic gene clusters. **(B)** Genome engineering applied to native natural product producing organisms can activate silent biosynthetic gene clusters through modification of promoter elements or the introduction of transcriptional activators. **(C)** Synthetic biology and DNA assembly technologies allow for the assembly of hybrid gene clusters to synthesize novel or modified natural product antibiotic drugs. **(D)** Natural product biosynthetic gene clusters can be transplanted from native producers into heterologous host organisms modified to maximize product biosynthesis. Figure constructed using Biorender.

Advances in genomics technology have allowed for a near exponential increase in the number of new microbial genome sequences (Medema and Fischbach, 2015). This wealth of genome sequence data, coupled with powerful computational search capabilities, has revealed new biosynthetic gene clusters that may potentially encode natural product biosynthetic pathways (Figure 1A), (Sekurova et al., 2019). However, a significant portion of newly discovered gene clusters are silent under laboratory conditions (Blin et al., 2019). This has led to extensive efforts to identify conditions that can activate silent natural product pathways in the native organisms in the hopes of discovering novel antibiotic molecules (Gehrke et al., 2019; Tomm et al., 2019). While it is likely that new antibiotic compounds will continue to be revealed by this strategy, it is an untargeted process whose outcomes are difficult to predict.

Synthetic biology can be described as the application of engineering principles to biological systems. The key power of synthetic biology lies in the ability to program cells to produce desired compounds or perform specific tasks. Therefore, synthetic biology and metabolic engineering can offer tools and means to reveal or to generate new antibiotic compounds. An early example of this strategy was the generation of hybrid gene clusters from distinct *Streptomyces* species to yield the production of novel natural product compounds (Hopwood et al., 1985). More contemporary applications of metabolic engineering to the production of antibiotics include the use of biosensors combined with mutagenesis to induce and detect activation of a silent gene cluster for the production of the antibiotic coelimycin in *Streptomyces lividans* (Sekurova et al., 2021). Further directed strategies have taken advantage of genetic and genomic data to express known or predicted transcriptional regulators to induce the expression of silent gene clusters encoding mayamycin, warkmycin and chartreusin-like compounds or engineer promoter elements to into a phenazine biosynthesis gene cluster to yield new derivatives of phenazine-1-carboxilic acid (Figure 1B) (Saleh et al., 2012; Mingyar et al., 2021). The ability to manipulate the individual gene components of biosynthetic gene clusters or generate hybrid proteins from closely related enzymes with distinct specificities also adds to the potential for engineering and mixing genetic sequence elements to generate novel small molecules with antibiotic potential, in effect accelerating evolution that might happen through naturally occurring gene transfer and/or genetic recombination events (Figure 1C) (Mao D. et al., 2018; Awakawa et al., 2018; Zhang et al., 2019).

An alternative to engineering the native producer of antimicrobial natural products is to transplant the biosynthetic gene clusters into heterologous production hosts (Figure 1D). These approaches have become possible through the application of recombination mediated engineering (recombineering) and transformation-associated recombination (TAR) strategies (Yamanaka et al., 2014; Li et al., 2015). Expression of heterologous biosynthetic gene clusters in favorable production hosts has been further enhanced by our ability to rapidly synthesize codon optimized open reading frame sequences. This has allowed for the assembly and optimal expression of complex natural product pathways in manipulable microbial host organisms like *Saccharomyces cerevisiae* (Ro et al., 2006). The recreation of complex pathways in heterologous production hosts bypasses the challenge of culturing and engineering poorly characterized microbes, and even allows pathway synthesis based upon metagenomic data (Wu et al., 2019).

### 2.1 Probiotics as Live Cell Therapeutics

The gut is a common site of infection by pathogenic bacteria and colonization can lead to serious disorders (Lamps, 2009). Infection and illnesses occur when a pathogen or toxins produced by a pathogen are ingested and the pathogen establishes itself within the host (Bintsis, 2017; Mousavi Khaneghah et al., 2020). Some notable bacterial pathogens include *Vibrio cholera, Salmonella*, *Clostridium* species, *Staphylococcus aureus*, *Shigella and Listeria*. Upon ingestion these pathogens can adhere to the mucosal layer and colonize the intestine (Martens et al., 2018). In some cases aggressive infectious microbes secrete toxins and enzymes that allow them to invade further into the epithelial layer where they can induce extensive tissue damage, inflammation and potentially gain access to other organs and the bloodstream (Fasano, 2002; Lamps, 2009).

Food-borne pathogens typically enter into a gut environment that has a fully established resident microbial community (Khan et al., 2021). Competition with the endogenous community, coupled with the host’s immune system, can often control infections. Indeed, some opportunistic pathogens are commonly resident in the gut of most mammals but do not trigger disease unless the endogenous microbial community is disrupted by stress or more often in response to antibiotic drugs that decimate the resident microbes creating an opportunity for pathogenic strains to rapidly expand (Raplee et al., 2021; Abd El-Hack et al., 2022).

Despite development in the areas of medicine, nutrition and food science, food-borne intestinal infections remain a global challenge. The World Health Organization (WHO) estimated that 22 foodborne pathogens caused about two billion illnesses, resulting in over one million deaths in 2015 alone (Kirk et al., 2015). The impacts of these illnesses are diverse and pose a significant threat to public health and socio-economic development globally. Since foodborne pathogens cause deaths and illnesses of millions of people worldwide, developing strategies to control and kill these microorganisms is a high priority. Antibiotic drugs have been our most powerful tool to prevent and treat pathogenic bacterial infection. However, antimicrobial resistant pathogenic bacteria have been reported with increased frequently and pose a challenge in the food industry. Therefore, there is an urgent need for alternative strategies to control foodborne pathogens and illnesses.

The gut environment is populated with commensal microbial species adapted with capabilities to thrive in a competitive environment. One strategy to combat invading pathogenic organisms is to harness and exploit beneficial microbial species. Probiotics are microbial organisms that safely reside in the gut and confer a health benefit to a host with potential for treatment of infectious diseases and inflammation of the gastrointestinal (GI) tract. Some of the most widely employed probiotic strains include *Lactobacillus*, *Bifidobacterium* and *Saccharomyces* species*.* These can survive the acidic environment of the stomach and colonize the gut to confer health benefits making them a staple throughout the food industry and offer the potential to act as live cell therapeutic agents (Mazzantini et al., 2021).

**FIGURE 2:**
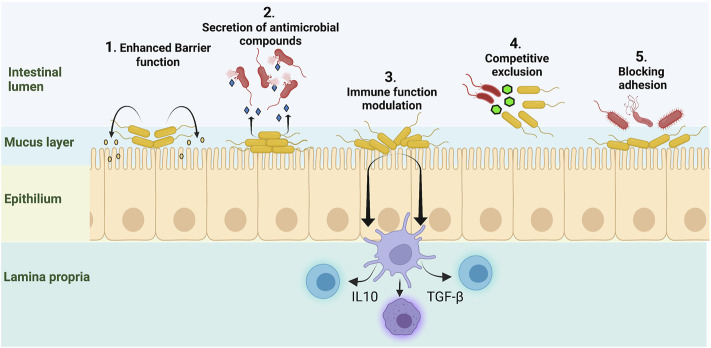
Native probiotic microbes provide beneficial activities. 1) Secretion of short chain fatty acids (SCFA) and other small molecules can enhance epithelial barrier function. 2) Probiotic strains can secrete anti-microbial peptides and other small molecules that inhibit pathogenic bacteria. 3) Probiotic strains can modulate inflammatory responses through influence on dendritic cells and T-cells to improve immune function and dampen secretion of inflammatory cytokines. 4) Probiotics compete with pathogens for space and nutrients thus limiting their proliferation in a crowded gut environment. 5) Resident microbiota and probiotics inhibit pathogen adhesion and colonization of the gut walls. Figure constructed using Biorender.

Probiotic microbes exhibit a diverse array of mechanisms that have potential to modulate the gut environment and immune system. Some of the benefits supplied by probiotic organisms are: 1) improvement of the intestinal epithelial layer barrier through secretion of metabolites and small molecules, 2) secretion of antimicrobial and anti-inflammatory factors, 3) ability to enhance innate immunity and modulate pathogen-induced inflammation. 4) competition with opportunistic pathogens for space and nutrients, and 5) inhibition of pathogen adhesion and translocation (Zhang et al., 2017; Wan et al., 2019; Flaugnatti et al., 2021) (Figure 2). However, these native abilities can be overwhelmed in response to dietary changes, stress, broad spectrum antibiotic treatments or other causes of dysbiosis (Gagliardi et al., 2018). Advances in genomics, DNA sequencing, DNA synthesis, DNA assembly techniques and probiotic research have provided the tools to engineer live cells therapeutics capable of addressing current challenges posed by disease causing bacteria.

### 2.2 Engineered Probiotics for Use in the Control of Foodborne Pathogens

**FIGURE 3:**
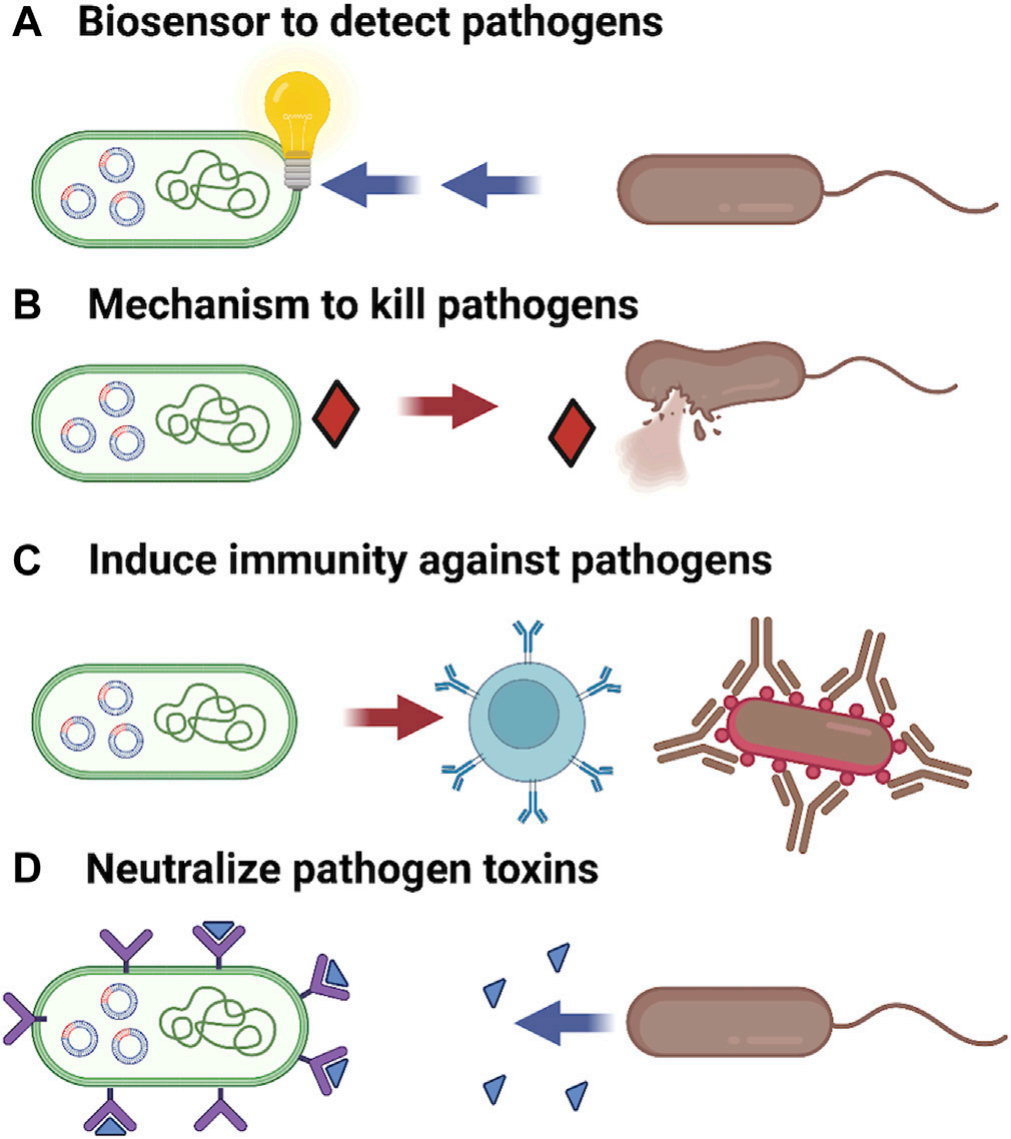
Probiotics can be engineered with improved functionalities to inhibit pathogens. **(A)** Receptors wired to a genetic circuit capable of providing an easily measurable response can act as biosensors to detect and even quantitate pathogens presence. **(B)** Secretion of bacteriocins and lytic enzymes can be programed into favorable strains to degrade biofilms and allowing specific killing of pathogenic microbes. **(C)** Engineering expression of pathogens surface markers on harmless probiotic strains can provide for vaccination to increase immunity against pathogens. **(D)** Expression of receptors for bacterial toxins can allow a probiotic to act as a biological vacuum to neutralize toxins and reduce tissue damage from infection. Additionally, probiotic strains can be programmed to secrete quorum sensor antagonists to reduce expression of virulence factors. Figure constructed using Biorender.

The power of synthetic biology lies in its ability to program living cells with functionalities that may not currently exist, or not exist in a context that can be applied to solve the desired problem. Synthetic biology in conjunction with adaptive laboratory evolution (ALE) strategies can be used to accelerate evolution and sample an increased chemical space that may be necessary to generate effective therapeutics to control antibiotic resistant microbial pathogens (Bober et al., 2018; Pedrolli et al., 2019). Among the possible opportunities for engineering live cell therapeutics include: 1) Building biosensors for rapid specific detection of pathogens. 2) Engineering probiotic strains with improved ability to compete with or specifically kill selected pathogens. 3) Rewiring metabolism in probiotic strains to modulate the host immune response to improve recognition of pathogens or reduce harmful inflammatory reactions. 4) Engineering probiotic strains to neutralize toxins secreted by pathogens or disrupt communication and virulence (Figure 3). It is notable that these are not mutually exclusive as strains can be engineered to include more than one of these functionalities. Here we will consider some recent advances that have been made in engineering probiotics for the treatment and prevention of foodborne illnesses induced by *Vibrio cholera*, *Staphylococcus aureus*, *Clostridium perfringens* and *Clostridioides difficile.*

#### 2.2.1 *Vibrio cholerae*

Cholera, a waterborne, life-threatening, gastrointestinal infection, is caused by the Gram-negative bacterial pathogen *Vibrio cholerae* and affects millions of individuals annually with an estimate of 21,000 to 143,000 deaths recorded worldwide (Zuckerman et al., 2007; Ali et al., 2015; Cho J. Y. et al., 2021)*.* It is characterized by watery diarrhea and vomiting which leads to rapid dehydration, hypovolemic shock, acidosis and death (Ali et al., 2015; Hsiao and Zhu, 2020). *V. cholerae* colonizes the epithelium of the small intestine where it can form a resistant biofilm, produces cholera toxin and toxin-coregulated pili and employs a type 6 secretion system to kill competing native gut bacteria (Faruque et al., 1998; Zhu and Mekalanos, 2003; Logan et al., 2018).

Cholera toxins are responsible for altering the hosts cellular signaling pathways causing cellular damage and the watery diarrhea characteristic of cholera; while the toxin-coregulated pili aids in colonization of the gut epithelium (Herrington et al., 1988; Cho J. Y. et al., 2021). Current treatments for cholera are centered around antibiotic therapy and while these have dramatically reduced case fatality, resistance to antibiotics has been demonstrated in cholera endemic and epidemic countries creating a need for new antimicrobial strategies (Clemens et al., 2017). Some natural probiotic microbes can aid in *V. cholerae* infections. For example, oral administration of *Lactobacillus acidophilus* and *Bfidobacterium* spp. in mouse models that were initially infected with cholera has shown to have enhanced mucosal and systemic immune response to the cholera toxin (Tejada-Simon et al., 1999).

Aside from antibiotic treatment, vaccination is considered key to controlling the spread of cholera (World Health Organization, 2018). Beyond the potential of their native therapeutic properties, probiotic microbial cells offer a potential vehicle for vaccine delivery to the gastric mucosa (Gardlik et al., 2012; Iqbal et al., 2021). The budding yeast *Saccharomyces boulardii* has properties including acid tolerance and good growth at 37°C that allow it to be employed as a probiotic (Sazawal et al., 2006; Czerucka et al., 2007; Kelesidis and Pothoulakis, 2012). This yeast has been engineered to express the *V. cholerae* toxin coregulated pilin gene tcpA, with the objective of inducing a pre-emptive immune response to inhibit gut colonization by *V. cholerae* (Awad et al., 2020). Although no data are available on whether ingestion of the probiotic can induce a strong mucosal immune response to *V. cholerae,* it is relevant that *S. boulardii* can be engineered to either secrete foreign antigens or display them on the cell surface (Wang et al., 2014; Li et al., 2021). Oral vaccination using recombinant probiotics as the delivery system could thus be an effective means to activate both the innate and adaptive immune responses of the host to reduce the risk of transmission of foodborne infections (Mathipa and Thantsha, 2017).

Many bacterial pathogens including *V. cholerae* employ a quorum sensing system (QS) to induce colonization, biofilm formation, virulence and broad dissemination in the host’s gastrointestinal tract (Hsiao and Zhu, 2020). QS is a signaling mechanism that bacteria use to respond to chemical hormone-like molecules called autoinducers, secreted by bacteria of the same species (Reading and Sperandio, 2006). The soluble QS molecule is of necessity unique to the pathogen and thus offers a target to allow detection of the pathogen. The *V. cholerae* quorum sensing system has been transplanted into *E. coli* and linked to a pathway to induce expression of a guide RNA that targeted dCAS9 to inhibit expression of a GFP reporter gene. In the absence of the *V. cholerae* quorum sensing molecule CA1, the GFP reporter was silenced. Upon binding of CA1 the expression of the gRNA was repressed leading to activation of the GFP reporter signal. This biosensor was extremely sensitive and allowed detection of *V. cholerae* (Holowko et al., 2016). The same group further engineered the *E. coli* biosensor and constructed a sense-and-kill suicide microbe (Jayaraman et al., 2017). Upon detection of the *V. cholerae* quorum sensing molecule a synthetic circuit was activated that induced expression of a synthetic endolysin that was exported to the periplasm leading to lysis of the biosensor and releasing the endolysin, leading to killing of the *V. cholerae* cells. A similar synthetic biosensor circuit was constructed in *Lactococcus lactis* which permitted effective detection of *V. cholerae* in infected mice (Mao N. et al., 2018). *L. lactis* has a natural capability to inhibit *V. cholerae* through secretion of lactic acid to acidify the local environment and consumption of a native strain is sufficient to reduce cholera-induced morbidity in mice (Mao N. et al., 2018). In this case the biosensor circuit was not linked to any mechanisms to kill or inhibit *V. cholerae* but this is a direction that would likely yield a powerful therapeutic tool (Mao N. et al., 2018). The power of such a synthetic circuit lies in the specificity of response, allowing the sense-and-kill strain to reside in the gut and only respond upon detection of the pathogen.

Signalling molecules of the quorum sensing pathway are critical to regulating the expression of toxins and other virulence genes by *V. cholerae*. These signaling molecules provide not only a biomarker to allow detection of the pathogen but provide a potential means to disrupt induction of virulence factors. An engineered *E. coli* strain expressing and secreting the *V. cholerae* autoinducer CA1 was able to disrupt toxin expression in mice inoculated with *V. cholerae,* leading to a significant increase in survival (Duan and March, 2010). An additional strategy to reduce the harm done by cholerae toxin has been to engineer a probiotic *E. coli* to express a surface receptor for cholera toxin. This probiotic could significantly reduce the amount of cholera toxin binding to the gut wall and improve recovery in a mouse model of *V. cholerae* infection (Focareta et al., 2006).

The concept of recombinant probiotics improving the inherent functions of natural probiotic strains, adding a surveillance attribute, being used as a drug or vaccine delivery system, or being “weaponized” to neutralize pathogen virulence factors, is a promising direction for the development of new therapies intervening with the pathogenic action of *V. cholerae*.

#### 2.2.2 *Staphylococcus aureus*

*Staphylococcus aureus* are a species of potentially pathogenic bacteria that are widespread in the environment. They commonly infect the blood, respiratory and most notably, the gastrointestinal tract of affected hosts. *Staphylococcus* infection is also one of the primary causes of community and hospital acquired infection, often resulting in severe and sometimes life-threatening conditions (Díaz et al., 2021). It is estimated that staphylococcal food poisoning causes approximately 241,188 illnesses and 1,064 hospitalizations in the United States alone (Scallan et al., 2011). *S. aureus* poses a serious public health hazard owing to the ability to secrete heat resistant enterotoxins that trigger inflammatory responses and the ability to form highly resistant biofilms that resist the host defenses, decrease the efficiency of antimicrobial factors, and provide resistance to antibiotics (Otto, 2013; Bintsis, 2017; Paik et al., 2019; Díaz et al., 2021). This pathogen infects a host and employs a quorum sensing signaling pathway to induce expression a collection of virulence factors including toxins, enzymes and secreted biofilm components that allow it to colonize and become established in the host.

The rapid appearance of antibiotic resistance in this microbe presents a challenge for developing antimicrobial therapies to treat and prevent infections. *S. aureus* acquires mutations at a rate that is about 1,000 times faster than for *E. coli* (Pipiya et al., 2020). This has allowed for the emergence of the notorious methicillin-resistant (MRSA) strains that are resistant to the entire β-lactam antibiotic class and created the need for improved therapies for detection and clearance of *S. aureus*. This is where bioengineered probiotics may have a pivotal role.

Several probiotic organisms including *lactobacillus* species, *Bacillus subtilis* and *S. boulardii* have demonstrated inhibitory activities toward *S. aurerus* though competitive exclusion, inhibiting growth by metabolite secretion, inhibition of biofilm formation and disruption of quorum sensing (Saidi et al., 2019; Nataraj and Mallappa, 2021). These antimicrobial activities coupled to the more general probiotic properties of reducing inflammation and improving epithelial barrier function make these organisms excellent candidates for engineering to improve upon their existing capabilities and to add more specific antimicrobial functionalities (Mathipa and Thantsha, 2017).

One way to improve the function of favorable probiotic strains is to make them more competitive within the gut environment. While these strains are all naturally tolerant of the acid conditions of the stomach and the routine 37°C temperature, one place that an advantage can be given is through bestowing resistance to the natural population of bacteriophage that populate the gut. Resistance to phage populations might permit a desirable probiotic strain to more effectively compete with pathogens and opportunistic pathogens that invade the human gut. Nagarajan *et al.* employed mutagenesis and a selective resistance procedure to isolate a probiotic bacterium, *Lactobacillus plantarum*, that was resistant to a phage population. *In vitro* competition tests between *S. aureus* and the selected *L. plantarum* strain demonstrated that in the presence of a phage population the probiotic was able to out compete *S. aureus* thereby reducing the ability of the pathogen to adhere to and colonize the epithelial cell layer (Nagarajan et al., 2019). This proof of principle experiment clearly has limited utility in that the probiotic was selected for resistance to the phage population from a specific environment and would likely be susceptible to phage from other environments. Additionally, given the high rate of mutagenesis in bacteriophage it is likely that phage capable of lysing the probiotic would readily emerge. None-the-less improving the competitiveness of favorable probiotic strains is a notion worthy of further investigation. Bacterial species with fully engineered and recoded genomes have been produced to eliminate redundant codons (Fredens et al., 2019). These are fully resistant to phage infection as they lack tRNA required for phage gene expression (Robertson et al., 2021). Application of this technology to a probiotic strain could give that strain a competitive advantage, allowing it to easily outgrow opportunistic pathogens in the gut.

Relatively simple genetic circuits have been engineered into probiotic strains to detect and in some cases respond to *S. aureus* infections. The probiotic bacteria strain *Lactobacillus reuteri* is effective in reducing *S. aureus* viability in a host through the mechanism of competitive exclusion, biosurfactant secretion and production of lactic acid (Sikorska and Smoragiewicz, 2013; Wang et al., 2015). An *L. reuteri* strain that expressed the *S. aureus* AgrA and AgrC genes to provide a receptor and response pathway for detection of the AIP1 signaling molecule was constructed such that activation of AgrA. would repress expression of a β-glucuronidase gene to allow colorimetric detection (Lubkowicz et al., 2018). This mimic system can detect the presence of the autoinducer at very low concentrations and could be useful for high-throughput quantitative and qualitative detection of *S. aureus*, especially in hospital settings (Lubkowicz et al., 2018). This method is also cost-effective in comparison to current mass spectrophotometry and DNA sequencing approaches. The probiotic system can also be fine-tuned as a chassis for the eradication of *S. aureus*.

Several probiotic organisms described above are capable of competing with *S. aureus* in the gut environment. In an effort to further improve the utility of probiotics to impeded *S. aureus* infections several groups have sought to “weaponize” probiotic strains through engineering them with genes encoding antimicrobial factors with specificity for the target pathogen. Bacteriocins are proteinaceous compounds produced by bacteria to facilitate anti-microbial activity against other bacteria (Bastos et al., 2010). Lysostaphin is a metallo-enzyme that is relatively specific for the cell wall structures of *Staphylococcal* species (Kokai-Kun et al., 2007; Bastos et al., 2010). *P. pastoris* strains that have been engineered to produce lysostaphin displayed efficient eradication of *S. aureus* during *in vitro* co-culture experiments (Pipiya et al., 2020). Despite the effectiveness of lysostaphin to kill *S. aureus in vitro* infections in the human gut pose a greater challenge owing to biofilm formation by the colonizing *S. aureus*.

A probiotic strategy to deal with *S. aureus* infections that has emerged is the use of engineered *Mycoplasma pneumoniae*. This organism has a small genome, is genetically manipulable, has an unusual codon usage and limited recombination that limits horizontal gene transfer (Osawa et al., 1990; Himmelreich et al., 1996; Krishnakumar et al., 2010; Sluijter et al., 2010). Additionally, it lacks a cell wall and so has a limited ability to trigger an inflammatory response in mammalian hosts (Sukhithasri et al., 2013). An *M. pneumoniae* with reduced virulence owing to deletion of the *mpn133* and *mpn372* genes was engineered to secrete dispersin B, a glycosyl hydrolase that can efficiently dissolve biofilms formed by *S. aureus*, and the bacteriocin lysostaphin (Garrido et al., 2021). The engineered strain was effective in disrupting and reducing *S. aureus* biofilm development in a catheterized mouse model as well as eliminating biofilms formed *ex-vivo* (Garrido et al., 2021).

*Staphylococcus aureus* remains a challenging pathogen for medicine to control owing to its ability to rapidly acquire drug resistance, the tenacious nature of its protective biofilms, ability to evade the immune system and ability to invade a variety of tissues. Clever genetic engineering of favorable host organisms to act as biosensors and “hunter-killers” that can disrupt biofilms may become a key tool that can be combined with other therapies including the use of engineered bacteriophage to combat this common microbial pathogen.

#### 2.2.3 *Clostridium perfringens*

*Clostridia* species are anaerobic organisms prevalent in soil. Several of these are well known human pathogens including *C. tetani, C. difficile and C. perfringens* and can induce disease in humans and agricultural animals (Popoff and Bouvet, 2009; Onderdonk and Garrett, 2020). The ability of these organisms to form spores that are resistant to heat and many common cleaning agents makes them challenging to control (Augustyn et al., 2022). *C. perfringens* is common within ecosystems and frequents the gastrointestinal tracts of livestock and poultry (Abudabos et al., 2019; Bai et al., 2020). It is responsible for a range of severe conditions, including food poisoning, gas gangrene, respiratory infections and necrotizing enteritis (NE) in both humans and animals (Lindström et al., 2011; Gervasi et al., 2014a). *C. perfringens* ranks second among the causes of foodborne illness and is responsible for greater than one million illnesses each year in the United States alone (Bintsis, 2017).

*C. perfringens infection* has been associated with the development of necrotizing enterocolitis (NEC), a gastrointestinal disorder affecting pre-term infants with a mortality rate of about 20% (Duchon et al., 2021). This pathogenic species is also the leading cause of necrotic enteritis (NE) in farm animals, costing the global poultry industry over two billion dollars annually, mainly due to the high prices of antibiotic treatments and therapeutic feed supplementation (Van Immerseel et al., 2009; Paiva et al., 2014; Smith et al., 2014). *C. perfringens*, like *S. aureus*, employs a quorum sensing system and secretes a battery of adhesins, proteolytic enzymes and toxins allowing it to colonize and invade the host (Pruteanu and Shanahan, 2013; Kiu and Hall, 2018; Navarro et al., 2018; Bai et al., 2020). They are also able to secrete bacteriocins that inhibit some competing bacteria to increase availability of nutrients and clear space to colonize (Mora et al., 2020).

Antibiotic drugs are the most common means to treat and prevent *C. perfringens* infection. However, the growing number of antibiotic resistant pathogenic strains found in isolated livestock and agricultural sources suggest their use may not be beneficial in the long term (Slavić et al., 2011). Indeed, up to 33% of isolates from some meat samples harbor *C. perfringens* with resistance to the common antibiotics: ampicillin, tetracyclin, amoxicillin, ciprofloxiacin and chloramphenicol. Further multidrug resistance was found in 38% of those isolates and multidrug resistant *C. perfringens* has been isolated from livestock (Zhang et al., 2021; Hassani et al., 2022). Vaccination processes that have been successful against *C. tetani* have also been developed for *C. perfringens* but have yet to become widely and successfully established (Alizadeh et al., 2021). The growing health and economic threat posed by resistant forms of *C. perfringens* is creating an urgent need to develop new approaches to controlling infection by this pathogen that are effective, affordable and can be widely disseminated.

The requirements for an effective chassis for a live cell therapeutic would include the ability to thrive in the gut environment, being genetically manipulable, controllable, exhibit limited horizontal gene transfer and have intrinsic properties that make it competitive to the pathogen. A variety of probiotic organisms including *Lactobacillus*, *Bifidobacterium*, *S. cerevisiae* and *S. boulardii*, have been demonstrated to compete with or inhibit the growth of *C. perfringens in vitro* and *in vivo* (Schoster et al., 2013; Guo et al., 2017; Chuang et al., 2019; Gong et al., 2020; Khalique et al., 2020; Wang et al., 2020). A limited number of probiotic species have been demonstrated to secrete bacteriocins that inhibit *C. perfringens* (O'Shea et al., 2009; Heo et al., 2018; Golneshin et al., 2020; Lee et al., 2021). These organisms and their bacteriocins may be exploited in the future as tools to inhibit opportunistic *C. perfringens* infections.

Extensive effort has been expended on engineering probiotic organisms to express *C. perfringens* virulence factors with the aim of generating effective live cell vaccines. Delivering toxins to humans or animals as a vaccine has inherent risks even if those toxins have been inactivated with heat or formaldehyde. In contrast, toxoids mimic toxins released by the pathogens but are not associated with virulence. A live cell expressing target antigens has the potential benefit of inducing immunity at the level of the gut mucosa and provides a mechanism that allows extended exposure to the toxoid antigen rather than single doses. Probiotic *Bacillus subtilis* has been engineered to produce, in cells, or display on spore walls, a fragment of *C. perfringens* toxin-α fused to gluthatione S-transferase. Mice immunized with toxoid displaying spores developed immunity and were protected against lethal doses of the toxin (Hoang et al., 2008). This approach provided immunity to only a single toxin but it could clearly be extended to cover other toxins. Additionally, the use of spores as a display and delivery vehicle provides a stable vaccine that could easily be produced in large volumes.

The probiotic bacteria *Lactobacilli casei* has been engineered to express a *C. perfringens* α-toxoid mimicking the α-toxin. Mice vaccinated with the probiotic developed anti-α toxin antibodies and survived challenge with a lethal dose α-toxin while the control animals rapidly succumbed to the effects of the toxin (Alimolaei et al., 2018; Song et al., 2018; Gao et al., 2019). Oral administration of an **ε**-toxoid expressing *L. casei* strain or a β-toxoid expressing *L. casei* strain was also able to induce an effective antibody response to the toxin (Alimolaei et al., 2016; Alimolaei et al., 2017). Similar results were found by Bai et al. and Zhao et al. who also engineered *L. casei* to express toxoids mimicking the *C. perfringens* α, ε, β1, and β2 toxins and vaccinated rabbits with the recombinant probiotic. Challenge experiments demonstrated that this strategy yielded an 80% protection rate, even more effective than vaccinating with inactivated, whole *C. perfringens* cells (Zhao et al., 2017; Bai et al., 2020). An attenuated *Salmonella typhimurium* expressing *C. perfringens* antigens NetB, α-toxoid and Fba administered to chickens induced both serum and mucosal antibody responses and provided partial protection from necrotizing enteritis intestinal lesions (Wilde et al., 2019). A similar investigation using a self-lysing *S. typhimurium* vector expressing α-toxoid and NetB administered to broiler chickens yielded induction of IgA, IgY, and IgM antibodies against the toxins and provided significant protection from a subsequent *C. perfringens* infection (Jiang et al., 2015). The yeast *S. boulardii* was engineered to secrete a fusion protein including the *C. perfringens* enterotoxin CPE. Oral administration of this live cell vaccine to mice induced both IgG and IgA antibody responses suggesting that *S. boulardii* may be an effective vector for application as a live cell vaccine (Bagherpour et al., 2018). Additionally, a “disarmed” *C. perfringens* strain lacking the NetB toxin, when orally administered to vaccinate broiler chickens, produced a significant protective effect and reduced the incidence of necrotizing enteritis lesions upon challenge with virulent *C. perfringens* (Mishra and Smyth, 2017). Recently, a native microbial strain isolated from poultry, *Ligilactobacillus agilis* La3, has been engineered to effectively produce *C. perfringens* NetB (Vezina et al., 2021). Although its effectiveness has yet to be characterized, it is likely that a native member of the poultry gut microbiota will be an effective delivery vehicle.

Metabolic engineering of probiotics has also been directed toward generating live cell therapeutics capable of not just competing with *C. perfringens* but able to attack and lyse *C. perfringens* to promote their clearance. This work has borrowed from the highly effective endolysin enzymes produced by bacteriophage to penetrate the cell walls of infected host cells to allow phage release (Schmelcher et al., 2012). Bacteriophage derived endolysins specific for binding to and degrading the cell walls of *C. perfringens* and have been identified and demonstrated to be effective for killing those organisms *in vitro* (Gervasi et al., 2014a). Additionally, protein engineering has been applied to improve activity and increase specificity of *C. perfringens* killing (Swift et al., 2015). *Clostridia* and other pathogenic bacteria can acquire resistance to antibiotic drugs through modification of or degradation of the drug or the use of efflux pumps. Endolysins specifically bind to and cleave highly conserved peptidoglycan structures that are essential to the integrity of the cell wall (Fischetti, 2010). Acquiring resistance to an endolysin would require the cells to develop changes in critical conserved cell wall structures that would likely reduce the integrity of that structure and so is expected to be a very rare event (Fischetti, 2006).

Synthetic biology techniques have been applied to engineer the probiotic bacteria, *Lactobacillus johnsonii,* secrete an endolysin against effective against *C. perfringens.* They found that this led to an improved ability of the probiotic bacteria to lyse *C. perfringens* cells and increased clearance of the pathogen in plate assays (Gervasi et al., 2014a; Gervasi et al., 2014b; Cho J.-H. et al., 2021). *S. cerevisiae* have been engineered to effectively display active *C. perfringens*-specific endolysins on their cell walls (Ritter and Hackel, 2019). These investigators used the yeast display system to engineer the endolysin for improved stability but they did not test them for *C. perfringens* killing. Such a system could be potentially employed with a probiotic yeast for application *in vivo*. While engineered *C. perfringens* killing probiotics have yet to be proven *in vivo* they display the potential to reduce pathogen load and aid in control of serious infections. Overall, these models demonstrate that probiotics can be manipulated to produce anti-microbial factors and directly promote the clearance of the pathogenic bacteria *C. perfringens*.

#### 2.2.4 *Clostridioides difficile*

*Clostridioides difficile*, like *C. perfringens*, is an anaerobic Gram positive bacteria that is widely disseminated in the environment. It is unclear how prevalent *C. difficile* is within the gut microbiome of human populations but it can be isolated from the gut of new born and healthy children (Tullus et al., 1989; Cui et al., 2021). *C. difficile* infection leads to diarrhea and colitis that can be fatal and is responsible for greater than 29,000 deaths each year in the United States (Lessa et al., 2015a; Lessa et al., 2015b). Indeed, *C. difficile* infection is among the top ten forms of health care associated infection and is a continuing problem in hospital environments (Suetens et al., 2018). *C. difficile*, like *C. perfringens*, secretes a set of toxins *tcdA* (toxin A), *tcdB* (toxin B), and *CDT* that are responsible for the tissue damage and pathology associated with infections (Kuehne et al., 2014; Chandrasekaran and Lacy, 2017). The pathology of *C. difficile* infection in humans and animals has been extensively reviewed (Abt et al., 2016; Moono et al., 2016).

Pathogenic infections with *C. difficile* are associated with conditions that yield dysbiosis including antibiotic treatments or severe stress that provide the opportunity for *C. difficile* to colonize the gut (Hooks and O'Malley, 2017; Vasilescu et al., 2021). The use of a subset of antibiotic drugs is associated with an increased risk of inducing *C. difficile* infections likely owing the reduction of competing microbial species in the gut caused by the treatment (Vardakas et al., 2016). Infections with *C. difficile* are notoriously persistent. Most antibiotic treatments are not successful and even following extensive antibiotic regimens infections are recurrent in 20%–40% of patients (Pepin et al., 2005; Vardakas et al., 2016). The high rate of recurrence may be due to the ability of the cells to sporulate and embed spores in the gut wall (Castro-Cordova et al., 2021). Owing to the persistent nature of *C. difficile* infections the antibiotics currently used in treatment are vancomycin, fidaxomicin and metronidazole (McDonald et al., 2018). An ominous trend is that these antibiotic treatments now display reduced effectiveness in treatment of *C. difficile*. The effectiveness of metronidazole has reduced from 95% to 75% since 2010. Even vancomycin has displayed a reduced effectiveness from 95% to 85% (McDonald et al., 2018). The increased incidence of recurrent *C. difficile* infections reflects a failure of the currently employed antibiotic treatments and reveals the need for more effective drugs or alternative therapeutic approaches (Marra et al., 2020; Sholeh et al., 2020).

There has long been evidence that the normal diversity and composition of the gut microbiome is able to suppress outgrowth of *C. difficile* and limit pathological colonization of the gut (Freter, 1955). Administration of probiotic microbes including *Bifidobacterium breve* and *Lacticaseibacillus casei*, *S. boulardii*, and *L. rhamnosus GG*, either alone or in combination with antibiotic regimens have seen some success in inhibiting or resolving *C. difficile* induced gut pathologies in animal models (Gorbach et al., 1987; McFarland et al., 1994; Esposito et al., 2021; Panpetch et al., 2021; Yang and Yang, 2021; Yang et al., 2022). Treatment with defined combinations of probiotic strains that compete with *C. difficile* for mucin as a nutrient source have shown some success (Pereira et al., 2020). More complex combinations of probiotic strains have reached stage III clinical trials for treatment of *C. difficile* induced pathology and the application of fecal transplantation is gaining acceptance as a treatment despite concerns over the need to characterize the donor microbiota (Khoruts et al., 2021; McGovern et al., 2021). The mechanism by which probiotic administration aids in resolving *C. difficile* infection is likely a combination of competition for resources coupled with the ability of a subset of microbiota to secrete secondary bile acids, bacteriocins, and other compounds inhibitory to *C. difficile* vegetative cells, germination of *C. difficile* spores and potentially disruption of *C. difficile* quorum sensing (Spinler et al., 2017; Thanissery et al., 2017; Lv et al., 2020; Gunaratnam et al., 2021).

The effective deployment of probiotic microbes to resolve and prevent *C. difficile* infection has motivated efforts to engineer probiotics for improved specific functions. The pathologies and morbidity associated with *C. difficile* infection are induced by the toxins *tcdA, tcdB*, and *CDT*. Monoclonal antibodies directed against the toxins have proven effective in limiting the pathology of *C. difficile* infection (Wilcox et al., 2017). But thus far vaccination with toxoids has had limited success at inducing immunity or treating *C. difficile* infection (Dieterle et al., 2019; de Bruyn et al., 2021) However, engineered microbial cells expressing *C. difficile* toxins can induce effective protective immune response when orally administered in animal models. An attenuated *V. cholerae* strain secreting a toxin A fragment fusion protein induced anti-toxin A antibody production and a protective immune response in mice that survived a subsequent challenge with *C. difficile* infection (Ryan et al., 1997). Engineered attenuated *Salmonella typhimurium* and *Bacillus subtilis* have both been used as vectors for secretion of toxin A fragments to demonstrate the induction of protective immune response through mucosal delivery of the vaccinating antigen (Ward et al., 1999; Permpoonpattana et al., 2011). Oral vaccination with a “disarmed” *C. difficile* engineered to express toxin fragments provided substantial protective immunity to *C. difficile* challenge in animal models (Donald et al., 2013; Wang et al., 2018). Similarly, administration of an engineered *Lactococcus lactis* expressing a fragment of toxin A, either secreted from the cells or displayed on the cell wall induced a strong immune response and provided substantial protection to *C. difficile* challenge in mice (Yang et al., 2013). Engineering *L*. *lactis* to express toxin A and toxin B fragments in combination provided no greater protection than toxin A alone (Guo et al., 2015). *Clostridia* employ surface proteins to assist with adherence to the intestinal cell wall and promote colonization. *L. casei* engineered to express and display the *C. difficile* SlpA on its cell wall were able to effectively engraft in mouse intestines following inoculation and triggered a robust immune response that protected hamsters from death following infection with *C. difficile* (Vedantam et al., 2018). Additionally, *C. difficile* surface antigen *CD0873* involved in attachment to the epithelial cell wall has emerged as an effective antigen for induction of a protective immune response and may be a good candidate for surface display on probiotics to induce immunity (Karyal et al., 2021). The apparent effectiveness of the live cell vaccines in animal models is encouraging and the relative ease with which a variety of toxin and surface antigens can be tested for the ability to induce protective immunity means that this approach may achieve further uptake.

One of the limitations of vaccination efforts against *C. difficile* is the relatively slow sero-conversion time as well as the inherent limitation of systemic antibodies directed against toxins in the intestinal lumen (Greenberg et al., 2012; de Bruyn et al., 2016). Microbial cells have been engineered to deliver therapeutic antibodies to neutralize *C. difficile* toxins during infection. *L. paracasei* strains were developed to express and secrete or display on their cell walls antibody chains capable of binding toxin B. Oral delivery of the recombinant probiotics provided protection against infection with a toxin B expressing *C. difficile* strain in a hamster model (Andersen et al., 2016). The yeast *S. boulardii* has been employed to express and secrete a multidomain neutralizing antibody directed against the major toxins of *C. difficile* (Chen et al., 2020). The engineered live cell therapeutic was effective in limiting disease caused by acute and recurrent infections by *C. difficile* in a mouse model. An advantage of a *S. boulardii* as a chassis for delivering antimicrobial activity is that it can be used in combination with antibiotic drugs that would render a bacterial probiotic ineffective. Additionally, this strategy has potential for further enhancement through improved expression and secretion of the therapeutic antibody. It could also be more broadly applied through expression of antibodies against other virulence factors.

A final consideration for deployment of live cell therapeutics to treat *C. difficile* infection is the application of lytic bacteriophage. Phage that specifically target *C. difficile* have been identified but treatment with phage has had limited success (Mayer et al., 2008; Meader et al., 2013; Nale et al., 2016; Whittle et al., 2022). The effectiveness of a *C. difficile*-specific phage was improved through engineering the phage to provide guide RNA to induce the endogenous *C. difficile* CRISPR system to target its own genome (Selle et al., 2020). Despite the improved effectiveness in killing *C. difficile* vegetative cells this phage will likely suffer the limitation of all phage therapies in that the target cells will rapidly evolve resistance.

Bacteriophage encoded endolysins with specificity for *C. difficile* cell walls have been identified (Mayer et al., 2008; Mondal et al., 2020). Oral application of purified endolysin has proven effective in controlling *C. difficile* infection in mice but owing to limitations of protein stability the therapeutic effect is modest (Peng et al., 2018). This limitation might be overcome through expression of the endolysin by a probiotic microbial strain allowing prolonged secretion into the gut and extending the time allowed for effective killing of colonizing *C. difficile*. While this is an intriguing idea it would be necessary to ensure that the endolysin did not have “off target” effects by killing other microbial species which might add to any dysbiosis rather than simply eradicate the pathogen.

The increasing frequency of drug resistant *C. difficile* infections and the severity of their pathology coupled with the high incidence of infections occurring in health care facilities creates urgency around the development of new strategies to control this pathogen. The research described above highlight the potential to use bioengineered probiotics as live cell vaccines and vehicles for delivering therapeutics to effectively control the damaging effects of opportunistic *C. difficile* outgrowth.

## 3 Emerging Applications and Challenges of Bioengineered Probiotics

Bioengineering can be used to enhance the overall efficacy and effectiveness of known probiotic strains, particularly in their application to antagonize and treat diseases. The previous sections discussed the ways by which genetic engineering could enhance the inherent therapeutic action of probiotics. Common approaches include: increasing the specificity of the probiotic microbes for their target molecules, improving the targeted therapeutic delivery action in infected hosts, and improving the direct antimicrobial activity of the probiotic against pathogenic infections of the gastrointestinal tract. Genetic modifications can also be applied to probiotic strains to improve tolerance of ecological or host environmental stress including high temperatures, acidification, oxygen and food processing.

### 3.1 Potential Benefits of Engineered Live Cell Therapeutics to Combat Food Borne Pathogens

Engineered probiotics have significant potential for deployment as tools to detect pathogenic bacteria and eliminate or control them within the gut environment. For this purpose, live cell therapeutics display a number of potential benefits.

**FIGURE 4:**
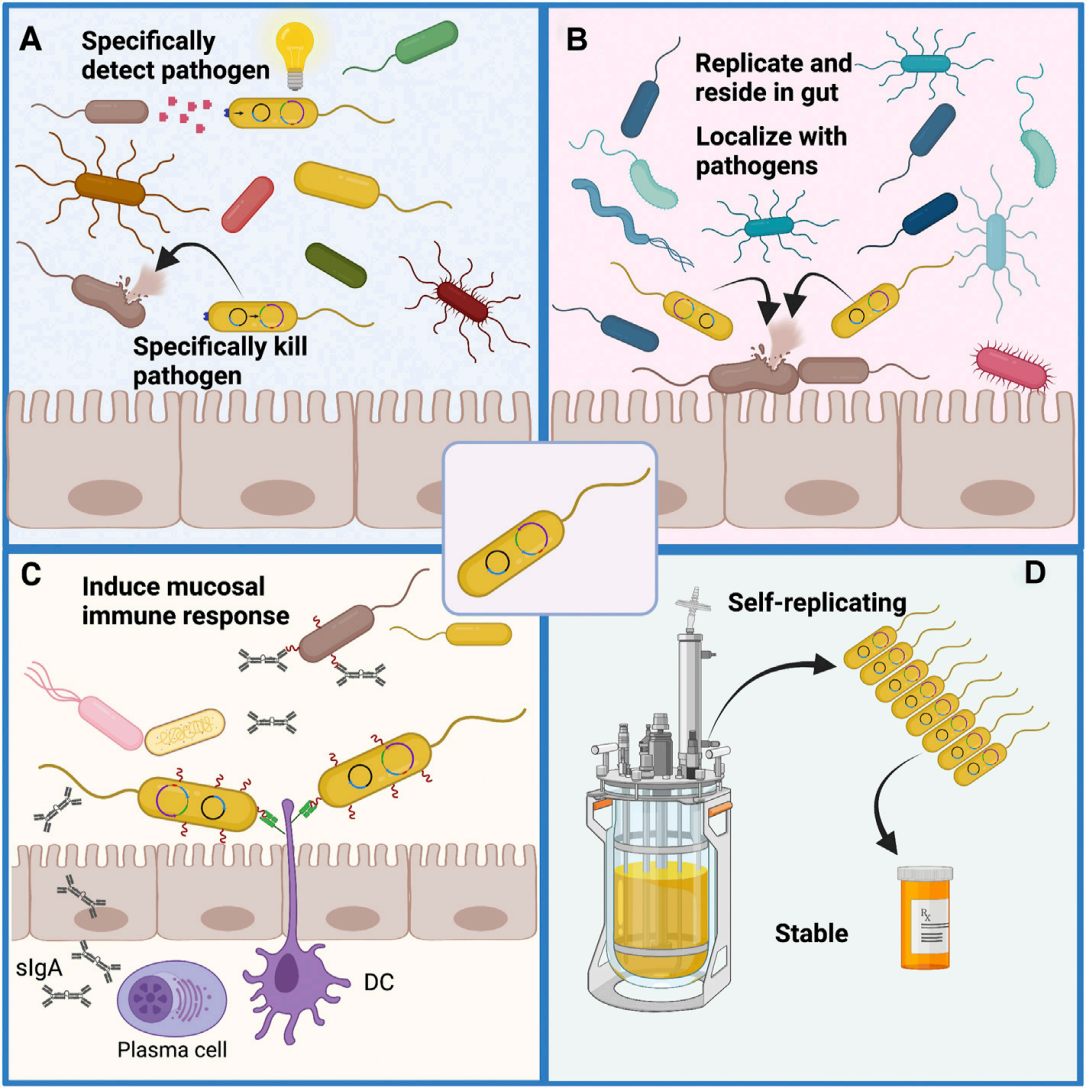
Potential benefits of engineered live cell therapeutics. **(A)** Engineered probiotics have the potential to be trained to detect specific pathogens by way of signaling molecules, surface antigens, secreted metabolites or other small molecules unique to the pathogen. This high specificity may allow for selective killing of the pathogen without loss of the resident microbial population, thus avoiding the dysbiosis that results from administration of broad-spectrum antibiotic drugs. **(B)** An engineered probiotic would reside in the gut in close proximity to sites of infection to allow direct and specific killing of intestinal pathogens with limited systemic effects. As the probiotic would self-replicate it would be effective for a prolonged time without the need for multiple applications. **(C)** As a live-cell vaccine, probiotics can act as a delivery vehicle to secrete or display pathogen surface antigens, adhesion molecules or toxin fragments to induce mucosal immunity at the site of infection which may have benefits over systemic immunization. **(D)** Engineered live-cell therapeutics can be produced in large quantity relatively cheaply through bioreactor culture and the cells can be stored dry or frozen with high viability.

1. Engineered probiotic strains that have been trained to detect and kill pathogens can have very high specificity for the target pathogen. These can be applied with precision to eliminate the pathogen without disruption of the gut microbiota, thus imposing less stress on the host and avoiding dysbiosis that would later need to be remediated (Figure 4A).
2. There is a low probability of engineered probiotics leading to “off target” effects. Unlike systemically delivered drug therapies, engineered probiotics are localized to the site of infection in the gut. Since these are known to be safe organisms they are unlikely to have adverse side effects (Figure 4B).
3. They do not generate or encourage the development of antibiotic resistance. Most antibiotic drugs are cytostatic, offering the opportunity for microbes to escape the drug. Probiotics can be engineered as cytolytic agents to kill the target pathogen, reducing the opportunity for developing resistance. Additionally, these will not spawn resistance to antibiotic drugs since they have distinct modes of action and in some cases such as engineered yeasts they could be deployed in conjunction with antibiotic drugs.
4. Live cell therapeutics with capability of killing pathogens will reside and replicate in the gut. Microbial cells can be engineered for increased stress resistance and improved ability to colonize the gut environment. Localized with the target pathogens, a live cell therapeutic is self-replicating and can remain in the gut to deliver its therapeutic activity for a longer period than any antibiotic drug would remain active, making it more effective with a lower number of administrations. In the absence of selection, the treatment is self-limiting as the therapeutic would be lost (Figure 4B).
5. As live cell vaccines, a probiotic expressing surface antigens or toxin fragments from a pathogen would have the advantage of generating mucosal immunity effective at the site of infection and colonization. Additionally, a live cell vaccine would allow for prolonged exposure to the target antigens rather than a single dose. An oral vaccine is simpler to provide and likely to have higher uptake than an injectable vaccine (Figure 4C).
6. Live cell therapeutics are self-replicating and so would be cheap to produce, transport and employ once developed. Although considerable time and money is necessary to generate highly effective live cell therapeutics, once generated, the production cost is low, it remains viable at room temperatures and can retain its stability in dried or frozen forms. This stability would permit their distribution in areas where prolonged freezing or refrigeration might not be practical (Figure 4D).

### 3.2 Challenges to Successful Application of Engineered Live Cell Therapeutics

Although bioengineered probiotics have been shown to be effective against pathogens that commonly infect the gut, their activity can be limited by several factors and their usage can also come with several challenges.

**FIGURE 5:**
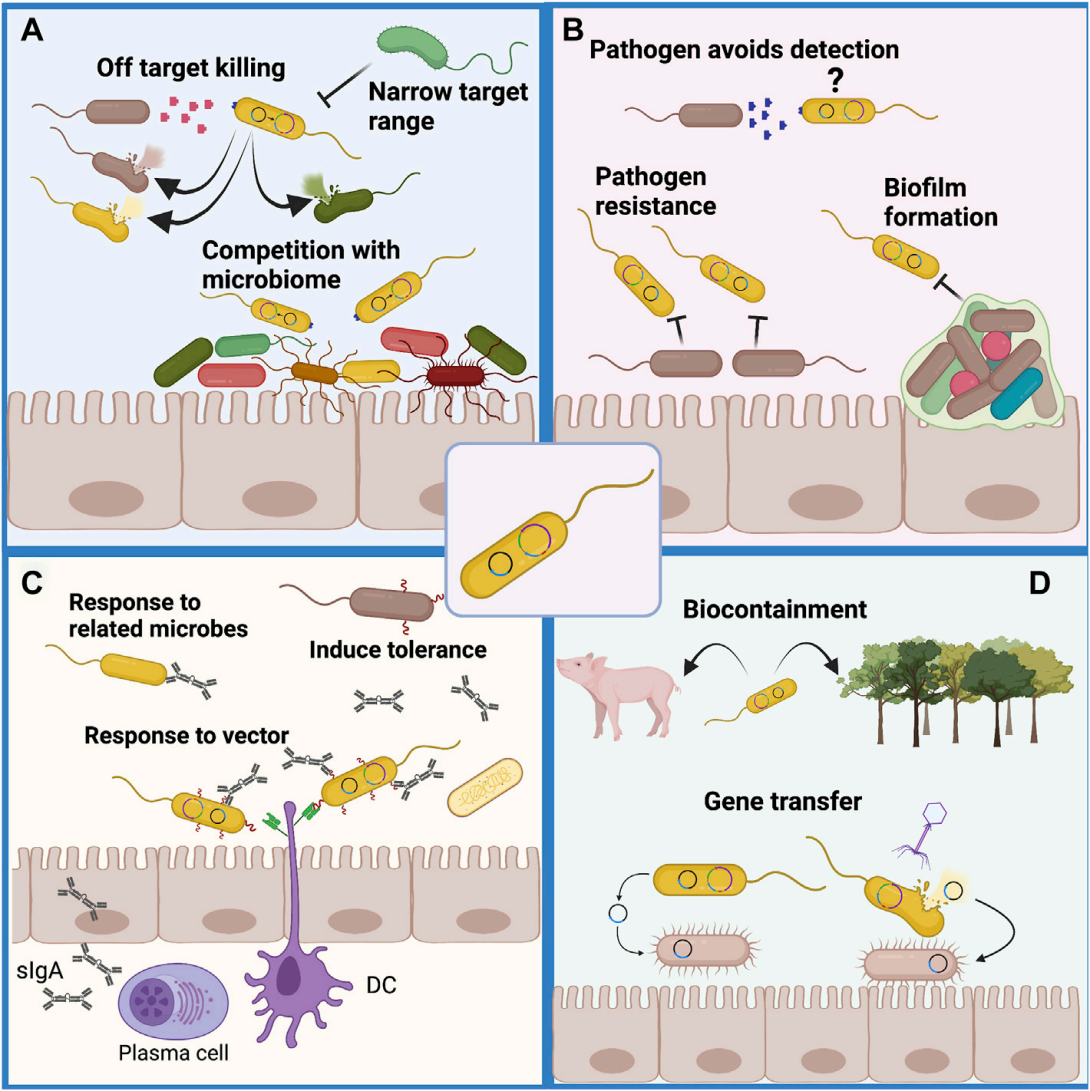
Challenges remain to the wide-spread application of engineered probiotics. **(A)** Engineered probiotics must compete with the resident microbial species for nutrients to replicate and then act on pathogens. To compete effectively they must be as well adapted as the resident microbes but must not contribute to overwhelming the resident microbiota. The killing of pathogens must be precise to avoid off-target killing of resident microbiota which would increase dysbiosis. This imposes the limitation that the pathogen must be specifically identified before the application of the engineered probiotic. **(B)** It is likely that variants of any pathogen will arise that can avoid detection or develop resistance to the mechanism of killing used by the engineered probiotic. Biofilms that are formed by a variety of pathogens that colonize the gut pose a challenge for both detection and killing of the pathogen. **(C)** Administration of live-cell probiotic vaccines presents the concern of tolerant pathogens resistant to immune cell responses from vaccination. Live cell probiotics can also be recognized by immune cells as foreign microbes and targeted for cell death, thereby decreasing their viability and vaccine actions. **(D)** Containment of an engineered probiotic is important as it is difficult to predict how the organism will act in different environments and there could be unexpected effects on animals, insects or plants for which the probiotic was not engineered. Even within the planned host environment transfer of genetic material to other organisms is a concern as this can occur through conjugation and direct transfer to bacteria as well as through phage mediated transduction and natural DNA uptake occurring upon cell lysis for any reason.

1. Competitiveness and stability within the host gut will be a challenge. The physical robustness of a probiotic microbe is critical to its ability to function as an antagonist against pathogenic bacteria (Sola-Oladokun et al., 2017). The human gut is populated by resident microbes well adapted to life in that environment. Any engineered organism will need to be genetically stable, survive passage through the GI tract, multiply and be highly competitive to have any hope of colonizing or engrafting in the gut. Expression of heterologous genes is likely to impose a significant metabolic burden on any engineered microbe making it less competitive within the gut environment. An engineered microbe might benefit from a “stripped down” genome engineered to remove non-essential sequences. This is likely to prove challenging since even *E. coli* has many genes and loci with no clearly assigned function (Ghatak et al., 2019). However, increased fitness for colonizing the gut environment could be a two-edged sword. If the engineered microbe is too effective it might displace the natural microbiota leading to unpredictable effects of dysbiosis (Figure 5A). They must also tolerate commercial scale production and storage process without contamination or significant loss of viability. A variety of strategies around this have been reviewed (Wan et al., 2019).
2. Target specificity needs to be tuned such that only pathogenic microbes are targeted and killed. Failure to achieve specificity for pathogens could lead to destruction of helpful microbial strains and increase dysbiosis (Figure 5A). Target specificity can be improved by means of sensors and switches to activate the killing mechanisms only when the target cells have been detected. Another approach might include expression of receptors on the surface of the engineered probiotic allowing it to adhere to or associate with the pathogen. However, while there is a benefit to the ability to target specific pathogens in the gut, this also presents the significant limitation that the target organism must be specifically identified (Figure 5A). In contrast, broad spectrum antibiotic drugs can be employed effectively even without specific characterization of the infectious organism.
3. Pathogen resistance will continue to pose a challenge. Even without specific selection, the diversity inherent in microbial populations means that variants that are resistant to the mechanism of killing programmed into a live cell therapeutic will arise (Figure 5B). It is also expected that pathogen variants with alterations in signaling molecules or molecules targeted by biosensors would eventually arise and avoid detection (Figure 5B). An additional means by which pathogens may develop resistance to any engineered probiotic is through biofilm formation (Figure 5B). While these can be overcome by production of degradative enzymes, they do pose a further challenge. Without selection these may not become prevalent but these will always limit the effectiveness of an engineered probiotic.
4. In the case of live cell vaccines, challenges will include achieving prolonged immunity following induction of mucosal immunity (Figure 5C). It is likely that this can be improved with suitable adjuvant strategies. Other concerns include the potential to induce tolerance to pathogens which might provide them increased opportunities. It is also possible that an immune response would be generated against the probiotic vehicle presenting the antigens. Such a response might reduce the effectiveness of the vaccine, induce a response to native gut microbiota leading to dysbiosis, and limit the effectiveness of readministering the live cell vaccine (Figure 5C). Any strains of the target pathogen with variation in the selected surface antigens may escape the immune response and induce the disease state.
5. Biocontainment: The importance of ensuring the safety of an engineered probiotic cannot be understated. While we may be able to demonstrate that a microbe has no negative effects within a lab environment or within test subjects, it is impossible to predict its behavior and interactions within other environments or in the guts of other organisms (Figure 5D). Auxotrophic requirements, self-limiting components and “kill-switches” can be engineered into live cell therapeutics to limit their dissemination but these may not be entirely sufficient for some purposes. Horizontal gene transfer can occur in most bacterial cell populations and this can be exacerbated through cell killing and by phage that can transduce genetic material (Figure 5D) (Sleator, 2010). In this regard, *S. cerevisiae* and *S. boulardii* have benefits as chassis organisms since they have no means for horizontal gene transfer, have no phage or virus that transmit cell to cell, and can be made sterile to avoid gene transfer to closely related wild yeasts.

## 4 Conclusion

The ominous rise in the frequency of infections caused by antibiotic resistant pathogens creates urgency around the development of new drugs and therapeutics to control infections. Synthetic biology and genomics offer powerful tools to discover, unmask and develop new natural product antibiotics and provide pipelines for their development. While this may prove to be a finite resource, it has yet to be fully exploited and may at least provide new compounds to keep us ahead of the wave of drug resistant pathogens. Engineered probiotics and live cell therapeutics are conceptually intriguing as they offer tools that can be applied with precision to detect, disarm and eliminate pathogens. While these solutions are provocative and exciting, a variety of challenges and concerns around safety and effectiveness must be overcome before this strategy can be considered a viable alternative to conventional therapies.
